# Molecular Epidemiology of *Salmonella enterica* Serotype Dublin Isolated from 2011 to 2022 from Veal and Dairy Cattle in Pennsylvania

**DOI:** 10.3390/microorganisms13020400

**Published:** 2025-02-12

**Authors:** Manoj K. Sekhwal, Lingling Li, Traci Pierre, Tammy Matthews, Erin Luley, Deepanker Tewari, Suresh V. Kuchipudi, Bhushan Jayarao, Maurice Byukusenge

**Affiliations:** 1 Penn State Animal Diagnostic Laboratory, Department of Veterinary and Biomedical Sciences, The Pennsylvania State University, University Park, PA 16802, USA; mmk6053@psu.edu (M.K.S.); lul17@psu.edu (L.L.); tap129@psu.edu (T.P.); tlm8@psu.edu (T.M.); bmj3@psu.edu (B.J.); 2 Bureau of Animal Health and Diagnostics, Pennsylvania Department of Agriculture, Harrisburg, PA 17110, USA; eluley@pa.gov; 3 Pennsylvania Veterinary Diagnostic Laboratory, Pennsylvania Department of Agriculture, Harrisburg, PA 17110, USA; dtewari@pa.gov; 4 Department of Infectious Diseases and Microbiology, School of Public Health, University of Pittsburgh, Pittsburgh, PA 15213, USA; skuchipudi@pitt.edu

**Keywords:** *Salmonella enterica* serotype Dublin, whole genome sequencing, core-genome sequence types, plasmids, histopathology, functional genes

## Abstract

The emergence of *Salmonella enterica* serotype Dublin (*S. Dublin*) presents significant challenges to animal and human health. We analyzed 109 *S. Dublin* isolates from bovine submissions to the Penn State Animal Diagnostic Laboratory between 2011 and 2022. Using whole genome sequencing, we assessed their phenotypic and genotypic resistance patterns and correlated these traits with case histories and pathology reports. Core-genome analysis identified cgSTs with similar allelic profiles between our isolates and those from the U.S. and Canada, while some cgSTs were unique to our study. Histopathologic findings suggest a predominance of respiratory and gastroenteric/hepatic lesions, aligning with the histopathological case definition for *S. Dublin* infection. Critically, all isolates were multidrug-resistant, particularly to ampicillin (87%), ceftiofur (89%), chlortetracycline (94%), oxytetracycline (94%), enrofloxacin (17%), florfenicol (94%), sulfadimethoxine (97%), and trimethoprim (20%). Plasmid genomic analysis unveiled distinct plasmid types including virulence, resistance, and hybrid plasmids, carrying unique compositions of virulence genes and antimicrobial resistance. These findings underscore the importance of managing calf movement to control the introduction and dissemination of new cgSTs in Pennsylvania and potentially nationwide. Furthermore, they emphasize the urgent need to mitigate *S. Dublin* transmission, combat antimicrobial resistance, and enhance surveillance efforts to effectively protect animal and human health.

## 1. Introduction

*Salmonella enterica* subspecies enterica serotype Dublin (*S.* Dublin) was detected in the western region of the United States as early as 1967, following which it spread to the Midwest and was isolated in 1980 in Indiana. By 1988, *S.* Dublin was found to be present in cattle in the eastern region of the United States, including New York, Ohio, and Pennsylvania [1]. Salmonellosis has been recognized as a significant disease in dairy cattle since at least the 1980s [2]. While its presence has long been established, its emergence as a multidrug-resistant (MDR) serotype has become a growing concern in last two decades across the United States [3,4]. A study examined data from U.S. surveillance systems, including the FoodNet database, and found that a higher percentage of human isolates of *S.* Dublin were resistant to more than seven classes of antimicrobial drugs during 2005–2013 (50.8%) than during 1996–2004 (2.4%) [4]. *S.* Dublin is considered a significant pathogen affecting dairy cattle worldwide. Reports from Great Britain and Canada indicate that at least 25% of cattle herds may be infected with *S.* Dublin [5,6]. Additionally, *S.* Dublin has been the predominant serotype identified among bovine *Salmonella* isolates from clinical samples submitted to veterinary diagnostic laboratories in both the U.S. and the U.K. [7,8,9]. In Germany, it ranks as the second most common serovar in registered salmonellosis outbreaks, contributing to 30–40% of cases [10]. Similarly, within the European Union, *S.* Dublin is the second most prevalent *Salmonella* serovar in cattle, following *S.* Typhimurium [11].

*S.* Dublin is host-adapted to bovines but has been infrequently reported in sheep, goats [12], and humans [9,11,13]. *S* Dublin is known to cause significant health issues in young calves. Studies have reported morbidity rates during outbreaks ranging from 10.5% to 34.8%, mortality rates between 2.3% and 18.2%, and case fatality rates of approximately 26.4% in dairy calves [14]. Many of these cases present as pneumonia and/or septicemia, while diarrhea [1] and abortion cases in infected herds [8] occur less frequently. There is significant consensus among researchers that cattle serve as the primary reservoir for *S. Dublin* and that antimicrobial-resistant strains originate from this reservoir. Furthermore, this reservoir could be a continuous source of multidrug-resistant (MDR) antimicrobial strains maintained through regular exposure to approved antimicrobial drugs to treat dairy, beef, and veal calves [14,15].

Advances in whole genome sequencing (WGS) over the last two decades have made it the best available method for characterizing microbe genomes. This technique helps us understand their evolution and diversity and provides valuable genetic information. Such information is key in developing effective public health strategies, including vaccines, antimicrobials, and prevention and control of infectious diseases. Hybrid genome assembly further enhances WGS by combining the accuracy of short-read sequencing with long-read sequencing, allowing for the reconstruction of highly contiguous and complete genomes. This approach is particularly valuable in resolving complex genome regions, such as those containing antimicrobial resistance genes, virulence genes, and mobile genetic elements, which are critical for understanding pathogen evolution and transmission [16]. Bioinformatics analysis is essential in interpreting the data generated through WGS and hybrid assemblies. Core-genome sequence typing (cgST) is a powerful technique that provides high-resolution typing for epidemiological tracking of bacterial strains. Unlike traditional MLST, which analyzes a small set of housekeeping genes, cgST examines hundreds to thousands of conserved core genes across the genome, offering much greater discriminatory power. This comprehensive approach allows for a more accurate identification of genetic differences between isolates, enabling researchers to trace the transmission pathways, outbreak sources, and evolutionary relationships of bacterial populations [17].

The focus of our study was to characterize *S.* Dublin isolates obtained from bovine submissions to the Penn State Animal Diagnostic Laboratory between 2011 and 2022. These isolates were analyzed for antimicrobial resistance/susceptibility and genotypically examined using WGS and bioinformatic analyses to determine core-genome sequence types (cgSTs). We applied a hybrid assembly approach to produce complete genomes, allowing us to explore molecular factors contributing to antimicrobial resistance, including resistance genes and mobile genetic elements such as conjugative plasmids, insertion sequences, and transposons.

In addition to characterizing the isolates, our study integrated pathological findings of necropsy cases and metadata related to the animals from which the bacteria were isolated. Although the isolates are not epidemiologically linked, combining phenotypic and genotypic data with contextual information such as case history, collection date, animal age, sex, type of farm (dairy, beef, veal), and pathology findings allowed us to identify patterns indicative of potential transmission. These findings are essential for designing targeted prevention and control strategies for *S*. Dublin in Pennsylvania and beyond.

## 2. Materials and Methods

### 2.1. S. Dublin Isolates

*S.* Dublin isolates (n = 109) from bovine submissions received at the Penn State Animal Diagnostic Laboratory from 2011 to 2022 were selected for the study. We selected all the samples that were submitted to the laboratory during this period. In 2010, the Penn State Animal Diagnostic Laboratory established a *Salmonella* repository isolated from cattle to characterize clonal types, virulence factors, and resistance to antimicrobials. The 109 *S*. Dublin isolates in this repository were from 109 cattle, including 72 veal calves and 37 dairy cattle (1 adult male, 8 adult dairy cows, and 28 female calves). The animals were from 17 dairy and 51 veal farms from 22 counties in Pennsylvania. Of the 109 isolates, 39 were from cattle submitted for necropsy to the Penn State Animal Diagnostic Laboratory, of which 18 and 21 isolates were male and female calves, respectively. Necropsy samples submitted for culture analysis included lung, intestines, rumen, abomasum, heart, liver, spleen, kidneys, lymph nodes, uterus, aborted fetuses, and placenta. The remainder of the isolates (n = 70) were from samples (blood, feces, field-necropsied tissues such as lung, liver, kidneys, spleen, placenta, and aborted fetuses) submitted by referring veterinarians for culture analysis.

These isolates were stored at −80 °C as a part of ADL’s *Salmonella* Culture Repository. Isolates from 2011 through 2018 were speciated using Sensititre Gram-negative identification plates following the manufacturer’s instructions (Thermofisher, Inc., Waltham, MA, USA). Plates were automatically read after 18 or 24 h and identified by the Sensititre SWIN software (Thermofisher, Inc.). From 2019 through 2022, Salmonella-presumptive-positive colonies were speciated using matrix-assisted laser desorption ionization mass spectrometry (MALDI-TOF; Bruker Daltonics, Fremont, CA, USA). Confirmed Salmonella isolates were serotyped at the National Veterinary Services Laboratories, Ames, IA, USA.

### 2.2. Review of Diagnostic Case Reports Associated with S. Dublin Isolates

On approval of the Pennsylvania Department of Agriculture Bureau of Animal Health and Diagnostic Services, the case reports associated with each of the 109 isolates underwent a process in which identifiers were removed. The study was limited to the following information: (1) date of submission, (2) reported age, (3) sex, (4) type of farm (dairy, beef, veal), (5) county in Pennsylvania, if provided, (6) gross and microscopic pathology of animals submitted for necropsy, (7) NVSL reported serotype, and (8) resistance to antimicrobials (MIC values).

Isolates (n = 27) that did not have antimicrobial susceptibility results were tested for antimicrobial susceptibility against 18 antimicrobial agents using the Sensititre BOPO6F plate (Thermofisher, Inc.), including ampicillin (AMP), ceftiofur (TIO), chlortetracycline (CTET), clindamycin (CLI), danofloxacin (DAN), enrofloxacin (ENRO), florfenicol (FFN), gentamicin (GEN), neomycin (NEO), oxytetracycline (OTET), penicillin (PEN), spectinomycin (SPE), tiamulin (TIA), Tilmicosin (TIL), Trimethoprim /sulfamethoxazole (SXT), tulathromycin (TUL), and Tylosin tartate (TYLT). Plates were read using the Sensititre Vizion System1 (Thermo Fisher), and minimum inhibitory concentrations (MIC) were interpreted using the Veterinary CLSI-defined (BOPO6F panel drugs) consensus breakpoints (CLSI, 2021 [18]).

### 2.3. Library Preparation and Sequencing

All strains of *S*. Dublin in this study were sequenced at the Penn State Animal Diagnostic Laboratory. The genomic DNA was extracted using the PureLink™ Genomic DNA Mini Kit (Thermofisher, Inc.) according to the manufacturer’s instructions. Two sequencing approaches were used: short-read sequencing with Illumina MiniSeq and long-read sequencing with Oxford Nanopore Technologies. All 109 isolates were sequenced using the Illumina MiniSeq, and 10 representative isolates were sequenced using Oxford Nanopore Technologies. The Illumina DNA Prep kit (Illumina, San Diego, CA, USA) was used to generate the libraries for sequencing with Illumina MiniSeq using a 300-cycle kit that generated paired-end reads of 150 bp in length. The one-dimensional (1D) native barcoding genomic DNA protocol (EXP-NBD104 and SQK-LSK109; ONT) was used to create libraries for sequencing with the MinION device. Base-calling and demultiplexing of reads from the MinION data were performed using MinKNOW v20.10 and EPI2ME v2020.2.10, respectively. We used unique barcode sequences assigned to each sample for demultiplexing, ensuring accurate read separation and downstream analysis.

### 2.4. Data Pre-Processing and Genome Assembly

The initial data quality assessments were conducted using FastQC v0.12.1 [19], and subsequently read quality enhancement and adapter trimming were conducted via BBDuk v38.96 [20], with parameters including trimq = 20 (average quality), qtrim = rl, and minlength = 50. Spades v3.15.4 [21] was used to assemble the genomes of all 109 isolates, and the quality of the resulting genome assemblies was assessed using Quast v5.2.0 [22].

Ten representative isolates were selected based on the different patterns of distribution of AMR genes. A de novo hybrid assembly approach was employed to achieve complete genome assemblies for the ten representative isolates among 109 isolates. The hybrid assembly pipeline for bacterial genomes implemented in Unicycler v0.4.8 [23] with default parameters was utilized. This approach takes advantage of the high accuracy of Illumina sequencing and the length of ONT sequencing to produce high-quality complete assemblies. Briefly, the pipeline starts with a de novo assembly using short reads followed by the alignment of long reads to the assembly graph, and then identification and trimming of sequence overlaps, as well as circularization and rotation of the genomes, commencing with the dnaA gene for chromosomes and repA for plasmids.

### 2.5. In Silico Typing of Isolates

*Salmonella* In Silico Typing Resource (SISTR) v1.1.1 [24] was used to confirm the species and serotypes of the isolates. Bioinformatics software and databases provided by the Center for Genomic Epidemiology (CGE) in their Bitbucket repository (https://bitbucket.org/genomicepidemiology/; accessed on 20 August 2023) were used to extract typing information from the whole genome sequence data. Plasmid sequences within the whole genome data were identified using PlasmidFinder v2.0.1 [25]. The MLST pipeline v2.0.9 [26] was used to determine the multilocus sequence types (MLST) from assembled genomes. MobileElementFinder v1.1.2 [27] facilitated the identification of mobile genetic elements (MGEs), and their association with plasmid genomes was investigated using blastn v2.13.0 [28]. The detection of antimicrobial resistance determinants was executed through AMRFinderPlus v3.10.24 [29]. In addition, Easyfig v3.0.0 [30] was used for linear representations of AMR genes associated with virulence genes and insertion sequence (IS) elements in the plasmid sequences.

The identification of core genome Multi-Locus Sequence Typing (cgMLST) was conducted using the cgMLSTFinder v1.2.0 (https://bitbucket.org/genomicepidemiology/cgmlstfinder/src/master/; accessed on 20 August 2023), a tool consisting of the EnteroBase Salmonella database [31] and the KMA algorithm v1.4.14 [32].

### 2.6. Acquisition and Processing of S. Dublin Data from NCBI SRA

To conduct a genomic comparative analysis, we accessed the NCBI Sequence Read Archive (SRA) database [33] on 19 August 2024 to find all records of isolates identified as *S.* Dublin (Supplementary Table S1). We filtered the dataset by removing records without geographical locations or collection dates in their metadata using the BioSample accession numbers associated with each SRA record. The raw reads for the selected records were downloaded using the fastq-dump command of the SRA Toolkit [34]. We processed the downloaded reads in the same way as the reads from isolates in this study.

### 2.7. Geospatial Mapping of Sample Sources

For most isolates, the case reports included information about the zip codes of the farms where samples originated. This study used these zip codes to map the sources of the isolates. ArcGIS Pro 3.3.1 (Esri, USA) was used for mapping. The shapefiles for the Pennsylvania counties and associated metadata were obtained from the Pennsylvania Spatial Data Access (PASDA) (https://www.pasda.psu.edu/; accessed on 9 September 2024).

## 3. Results

### 3.1. Genomic Diversity and Temporal Distribution of Isolates from Pennsylvania

**Figure 1:**
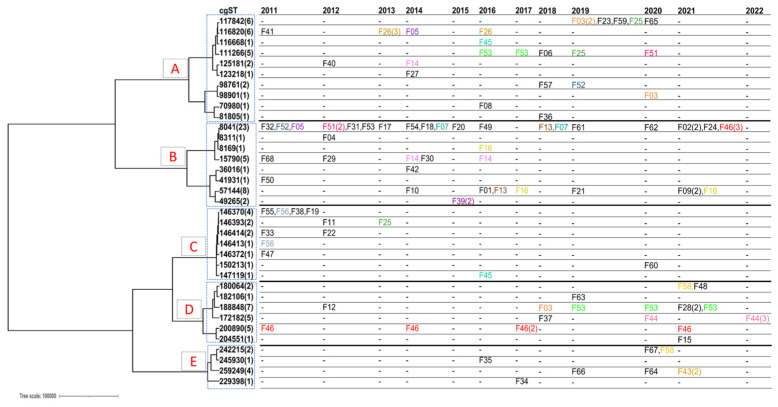
Distribution of *Salmonella* Dublin among veal and dairy isolates in Pennsylvania (2011–2022). The isolates are grouped into phylo-clusters (A–E) based on allele distance, with the total number of isolates in each cluster indicated in parentheses. A minimum spanning tree is used to visualize the allele differences between cgSTs. Non-black colors represent farms that either share one or more cgSTs with other farms or have the same cgST detected across multiple years.

A total of 109 *S.* Dublin isolates collected from 2011 to 2022 were obtained from necropsy cases and diagnostic samples submitted to the Penn State Animal Diagnostic Lab. These samples came from 109 cattle on 68 farms, including 17 dairy and 51 veal farms, spanning 22 counties in Pennsylvania. All isolates belonged to sequence type (ST) ST10 of the *Salmonella* MLST scheme and were classified into 35 core genome sequence types (cgSTs). Twelve cgSTs (96871, 111266, 116820, 117842, 8041, 15790, 57144, 146370, 146393, 172182, 188848, 259249) were found in both veal and dairy cattle, accounting for 71% of isolates. In comparison, 20 cgSTs were observed only in veal calves, while 3 cgSTs (204551, 200890, 150213) were observed in only dairy cattle (Supplementary Table S2). The 35 cgSTs were placed in five phylo-clusters (A–E) based on the allelic distances between cgSTs. These five clusters were analyzed for time (year) and source (farm) (Figure 1).

Cluster A included ten cgSTs, four of which (98761, 111266, 116820, and 117842) were found in both veal and dairy cattle. Among these, cgST 111266 and 116820 were prevalent over multiple years. These four cgSTs accounted for 73% of the isolates in this cluster. Meanwhile, cgSTs 98761 (2018, 2019) and 117842 (2019, 2020) occurred infrequently in veal and dairy cattle, respectively. The remaining six cgSTs were observed in veal calves and occurred infrequently over the ten years (2012, 2014, 2016, 2018, and 2020). Except for two cgSTs (116820, 125181), the remaining eight cgSTs were more prevalent after 2014 through 2020. The cgSTs in this cluster were not seen in 2021 and 2022. The frequency of occurrence of these ten cgSTs ranged from one to four cgSTs/year and one to five farms/year.

cgST 70980 was isolated from veal calves in Pennsylvania in 2016. cgST 111266 was observed in cattle from the same dairy herd for two consecutive years (2016 and 2017) and was later detected in veal calves on three different farms from 2018 to 2020. cgST 116668 was isolated from veal calves in 2016, while cgST 116820 was isolated from the same veal farm in 2011, 2013, 2014, and 2016. cgST 117842 was first observed in a dairy calf in 2019 and later in 2020 in veal calves on four farms from four different counties. cgST 123218 was observed from a veal calf in 2014, and cgST 125181 was isolated in veal calves from two veal farms in 2012 and 2014 (Figure 1; Supplementary Table S2).

Eight cgSTs belonged to cluster B. Three of them (8041, 15790, and 57144) were prevalent over multiple years in both veal and dairy cattle and accounted for 86% of the isolates. The remaining five cgSTs (cgST 8169, 8311, 36016, 41931, and 49265) were detected only in veal calves. A key finding related to the distribution of the cgSTs associated with this cluster is that cgST 8041 was the most prevalent, from 2011 to 2016 and again from 2018 to 2021, in veal calves and dairy cattle on 18 farms, including 5 dairy herds and 13 veal facilities in 12 counties in Pennsylvania (Figure 1; Supplementary Table S2).

Cluster C included seven cgSTs, two of which (146370 and 146393) were detected in 2011, 2012, and 2013 in both veal and dairy cattle. Four cgSTs (146372, 146413, 146414, and 147119) were observed in veal calves, while cgST 150213 was detected in dairy cattle in 2020. cgSTs 147119 and 150213 were observed in veal calves (2016) and in dairy cattle (2020), respectively, while the other five cgSTs were detected between 2011 and 2013. Notably, cgST 146370 was observed in 2011 in veal calves on three farms and once in dairy cattle (Figure 1; Supplementary Table S2).

Cluster D consisted of six cgSTs, among which cgST 172182 was observed in 2018, 2020, and 2022, while cgST 188848 persisted from 2018 to 2022. Both cgSTs were identified in veal and dairy cattle. cgSTs 182106 and 180064 were observed in veal calves in 2019 and 2021, respectively. All the cgSTs except for 200890 might have appeared after 2017 and continued to persist through 2022, while cgST 200890 was first seen in 2011 and later found in the same herd in 2014, 2017, and 2021.

Cluster E comprised four cgSTs, all detected in Pennsylvania after 2016. cgST 259249 was detected in veal and dairy cattle in 2019, 2020, and 2021, while the other three cgSTs were observed only in veal calves (Figure 1; Supplementary Table S2).

### 3.2. Geospatial Distribution and Global Contextualization of S. Dublin Isolates from Pennsylvania

**Figure 2:**
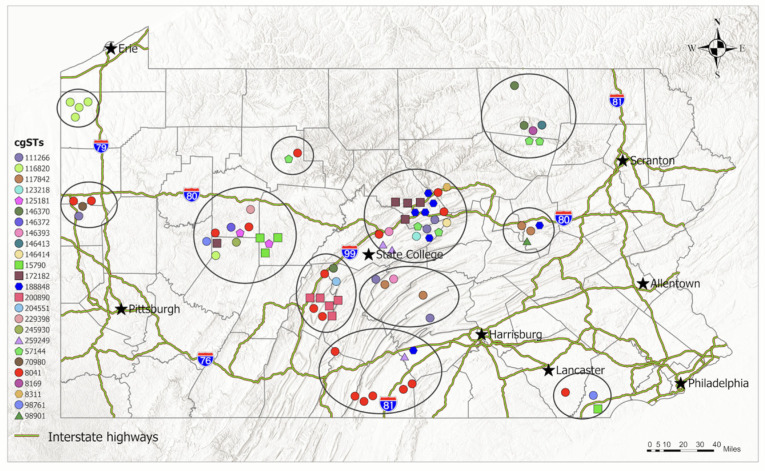
Geolocation of sources of *S.* Dublin isolates in Pennsylvania from 2011 to 2022. The shape–color label combination represents cgSTs of 81 isolates for which geographic information was available. The Disperse Markers tool of ArcGIS Pro was used to display points that are too close to one another. The gray circles show identified geo-clusters.

The spatial analysis showed that the sources of isolates form geo-clusters along the interstate highways (Figure 2). There appears to be greater diversity in clusters located in central counties compared to those in the peripheral counties. We observed a localized distribution of cgSTs, with most present in only one or two geo-clusters. Three cgSTs (57144, 111266, 188848) were found in three different geo-clusters. Exceptionally, cgST 8041 had the widest distribution among all the cgSTs in this study, being present in seven different geo-clusters (Figure 2).

To contextualize the *S.* Dublin isolates from Pennsylvania within a broader epidemiological framework, we analyzed cgSTs from publicly available genomic data in the NCBI SRA database. A total of 4485 records of *S.* Dublin were retrieved. After filtering out records lacking metadata on geographic location or isolation date and those with poor quality, 3814 records remained. Among these, only 118 records, all from North America (USA and Canada), exhibited cgSTs identical to those of the 109 isolates sequenced in this study.

Similar to the cgSTs in our study, those from public datasets also showed variations in their temporal and geographical distribution across different states of the USA and Canada. Below is a description of isolation locations, dates, and sources for these records. Supplementary Table S1 lists their corresponding SRA accession numbers.

Three cgSTs (98761, 98901, and 116820) in phylo-cluster A were only seen in Pennsylvania in this study. Some cgSTs were seen in data from multiple states, while others were found in only one other state. cgST 117842 was detected in a human blood sample in the U.S. (2018), in beef from Wisconsin (2022), in a farm environment in New York (2023), and in cattle in Canada (2023). cgST 125181 was detected from a dog treat in Colorado (2015), cattle in Ohio (2017), and ground beef from West Virginia (2023). cgST 81805 was isolated from cattle in Iowa (2015) and Wisconsin (2022), while cgST 116668 was observed in cattle from Ohio (2012 and 2015) and Wisconsin (2023). Other cgSTS that were seen in only one other state include cgST 70980 from beef in New York (2017), cgST 111266 from raw beef in Wisconsin (2023), and cgST 123218 from cattle in Ohio (2014) (Supplementary Table S1).

The cgSTs in the phylo-cluster B cluster were also found in SRA records from several other states in the U.S. and Canada. cgST 8041 seems to have appeared around the same time in 2010–2012 in New York and in veal and dairy cattle in Pennsylvania (Supplementary Tables S1 and S2). cgST 8041 was also identified in 2015 and 2016 from cattle in Iowa and Canada (2022) and in beef from Ohio (2023) and Pennsylvania (2024). cgST 8169 was isolated from cattle in New York in 2012. cgST 15790 was this study’s most widely distributed cgST, detected in seven states. It was identified from ground beef and cattle in Idaho (2019 and 2022), dairy cattle in Wisconsin (2003, 2022, 2023), and dairy cattle (2015) and ground beef (2018) in Minnesota. This cgST was also frequently observed in comminuted beef from Texas (2023), California (2018, 2021, 2023, 2024), Washington state (2023), and Nebraska (2023). cgST 36016 was isolated from comminuted beef in Tennessee (2021), cattle in South Dakota (2014), and joint fluid from humans in Canada (2017). cgST 57144 was observed in isolates recovered from human blood and urine samples in the U.S. (2016 and 2017) and in cattle in Canada (2022 and 2023).

Of the cgSTs of the phylo-cluster C, only one cgST 146370 was identified in SRA records from other states in the U.S. It was detected in comminuted beef from Nebraska (2022) and Texas (2023) and cattle from Canada (2023).

The phylo-cluster D comprised the most recent cgSTs, with most of them being isolated after 2017. cgST 172182 was found in SRA data from Pennsylvania in 2023. cgST 180064 was detected in cattle in Minnesota (2018) and Ohio (2024) and from a dog in Pennsylvania (2023). cgST 182106 was detected in raw pork in Wisconsin (2019), while cgST 200890 was isolated from an environmental sample in Ohio (2019). cgSTs 204551 was isolated in cattle from New York in 2019 and 2023.

Four cgSTS of the phylo-cluster E are also relatively recent and were identified in data from three other U.S. states and Canada. cgST 229398 was isolated from a rodent in New York (2019) and a beef sample from Wisconsin (2021). cgST 242215 was isolated from comminuted beef in Minnesota (2020), cgST 245930 from a human sample in Canada (2015), and cgST 259249 in cattle from Pennsylvania (2020) (Supplementary Table S1).

### 3.3. Pathology Findings from Necropsy Cases

Eighteen male calves were identified as positive for *S.* Dublin following necropsy. These calves were received for necropsy over ten years from 14 farms in Pennsylvania. The age of the calves ranged from 14 to 252 days, with a mean and median of 80 and 59 days, respectively. The respiratory system was most often affected, with lesions in 89% of calves. The pattern of lesions was characterized as either bronchiolar, interstitial, or mixed. Bronchopneumonia, defined by inflammatory cells (most often neutrophils) within bronchioles and alveoli with epithelial damage, was present in 34% of cases with respiratory lesions. Interstitial pneumonia, characterized by inflammatory cells within interstitial tissues and/or necrosis of these tissues, was present in 28%, and a mixed pattern showing features of both was also present in 28% of calves (Table 1).

Gastrointestinal and hepatic lesions were identified in 61% and 55.5% of the calves, respectively. Typical lesions included acute necrosis of the intestinal mucosa with infiltration of neutrophils and sloughing of the mucosa, with varying degrees of submucosal or transmural inflammation. They involved the small intestine, cecum, and colon. Inflammation of the abomasal mucosa and abomasal ulcers were present in 5.5% and 5.5% of calves, respectively. Overall, 50% of the calves exhibited enteritis, while 11% had peritonitis. Hepatic lesions were characterized as multifocal to coalescing, often randomly distributed, and necrosis with inflammation, which occurred in 50% of the calves, while 5.5% showed hepatomegaly. Overall, 50% of cases had lymphoreticular-involvement tissues, including 22% of the calves with splenic lesions characterized by multifocal suppurative necrosis, and 39% of calves had lymph node lesions, predominantly diffuse infiltration of neutrophils (suppurative lymphadenitis). Renal lesions were present in 28% of the calves and were described as tubulointerstitial nephritis (17%) and interstitial nephritis (11%). A solitary case (5.5%) of pericardial effusion was observed (Table 1).

Over nine years, 21 female dairy cattle from 13 farms were identified as positive for *S*. Dublin following necropsy. The ages of the animals ranged from 21 to 630 days, with a mean and median of 116 and 84 days, respectively. The respiratory system was most often affected, with lesions present in 95% of the animals. The pattern of lesions was characterized as either bronchiolar, interstitial, or mixed. Bronchopneumonia was present in 24% of the cases, interstitial pneumonia was present in 29%, and a mixed pattern showing features of both was also present in 43% of animals (Table 1). Gastrointestinal and hepatic lesions were identified in 80% and 61% of the animals, respectively. Inflammation of the abomasal mucosa and abomasal ulcers were present in 14% and 5% of animals, respectively. Overall, 52% of the animals exhibited enteritis, while 5% of each of the animals had peritonitis and rumenitis. Hepatic lesions were identified in 61% of animals as hepatic necrosis and 43% of cases had lymphoreticular involvement, with 24% of cases displaying splenic lesions and 24% with lymphadenitis. Renal lesions were present in 28% of the calves and were identified as tubulointerstitial nephritis (19%) and interstitial nephritis (10%). A 630-day-old female had cardiomyocyte degeneration (5%) and placental necrosis (5%) (Table 1).

### 3.4. Phenotypic and Genotypic Patterns of Antimicrobial Resistance

Results of antimicrobial susceptibility testing could not be interpreted for nine antimicrobials (clindamycin, danofloxacin, neomycin, penicillin, spectinomycin, tiamulin, tilmicosin, tulathromycin, and tylosin), as MIC interpretative criteria have not been established by CLSI (2021) [18] for Enterobacteriaceae. However, MIC interpretative criteria were available for nine antimicrobials, including ampicillin, ceftiofur, chlortetracycline, enrofloxacin, florfenicol, gentamicin, oxytetracycline, sulfadimethoxine, and trimethoprim/sulfamethoxazole. A high proportion of the isolates were resistant to ampicillin (87%), ceftiofur (81%), chlortetracycline (94%), florfenicol (94%), oxytetracycline (94%), and sulfadimethoxine (97%). Resistance to enrofloxacin, gentamicin, and trimethoprim/sulfamethoxazole was observed in 17, 0, and 20% of the isolates, respectively (Table 2).

Antimicrobial resistance gene *bla*_CMY-2_, which confers resistance to beta-lactams (ampicillin and ceftiofur), was observed in 92% of the isolates. Overall, 25% of the isolates had a truncated version of the *bla*_TEM-1B_ gene along with *bla*_CMY-2_. At the same time, 87% of the isolates were phenotypically resistant to beta-lactams with MIC values greater than 16 µg/mL. The *tetA* gene, which confers resistance to tetracyclines (chlortetracycline and oxytetracycline), was present in 96% of the isolates. In total, 94% of the isolates demonstrated phenotypic resistance to tetracyclines with MIC values greater than 8 µg/mL. Three variants of the *gyrA gene—gyrA-*S83Y (5%), *gyrA*-D87Y (2%), and *gyrA*-S83F (3%) that confer resistance to quinolones (e.g., enrofloxacin) were also detected, and 17% of isolates showed resistance to enrofloxacin (Table 2).

Subclasses of aminoglycoside O-phosphotransferases, *aph*(3′)-Ia and *aph(3′′)*-Ib, which confer resistance to aminoglycosides such as kanamycin, were detected in 15% and 96% of the isolates, respectively. However, all of the isolates were sensitive to gentamicin. The sulfonamide resistance gene *sul*2 was present in 96% of the isolates, with 97% having MIC values greater than 256 µg/mL for sulfadimethoxine. No trimethoprim resistance gene was detected (Table 2).

**Figure 3:**
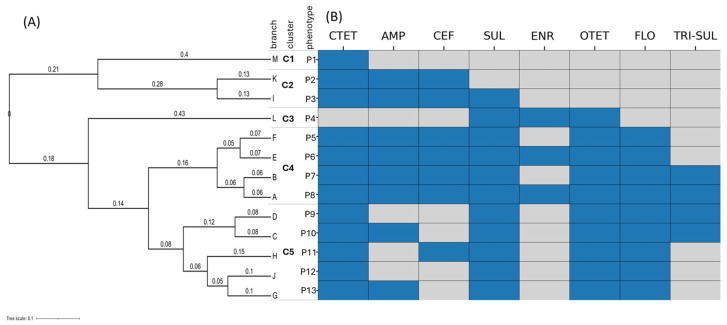
Phylogenetic clustering of *S.* Dublin isolates based on antimicrobial resistance (AMR) phenotypic profiles from veal and dairy cattle in Pennsylvania (2011–2022). (**A**) The dendrogram represents hierarchical clustering of isolates, with branch lengths indicating the genetic distance calculated based on the binary presence (1) or absence (0) of resistance genes. (**B**) The heatmap displays corresponding phenotypic resistance patterns (P1–P13), where blue cells indicate resistance to specific antimicrobial classes. AMP (ampicillin), ceftiofur (CEF), chlortetracycline (CTET), enrofloxacin (ENR), florfenicol (FLO), oxytetracycline (OTET), sulfadimethoxine (SUL), and trimethoprim/sulfamethoxazole (TRI-SUL).

**Figure 4:**
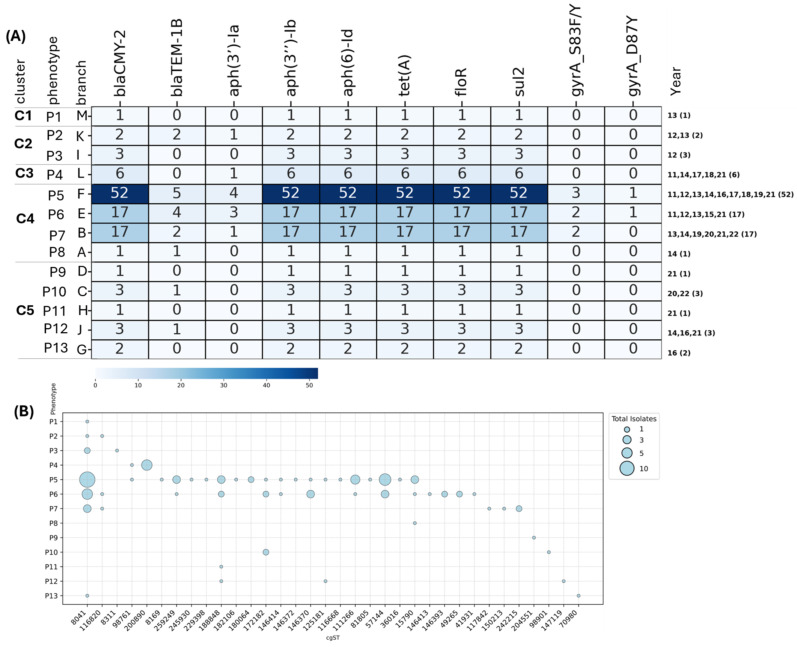
Distribution of *S.* Dublin antimicrobial resistance (AMR) genotype profiles across different years. (**A**) The heatmap illustrates the presence of AMR genes in isolates, with darker shades indicating a higher frequency of occurrence. The numbers in brackets represent the total count of isolates exhibiting specific resistance profiles. (**B**) The bubble plot displays the distribution of isolates based on their cgST across different years, with bubble sizes corresponding to the total number of isolates.

Isolates resistant to the nine antimicrobials were analyzed to determine the genotype and phenotype antimicrobial resistance profiles (Figure 3 and Figure 4). A total of 13 antimicrobial resistance phenotypes (P1–P13) were observed, with 108 out of 109 (99%) isolates being multidrug-resistant (resistant to more than three antimicrobials). These phenotypes were grouped into five clusters.

The first cluster comprised a single isolate (branch M), which exhibited five AMR genes and phenotypic resistance to chlortetracycline. This isolate belonged to cgST 8041 from one herd from 2013 (Figure 4A).

The second cluster (branches K and I) contained five isolates. All five isolates were resistant to ampicillin, ceftiofur, and chlortetracycline, with one isolate also resistant to sulfadimethoxine (Figure 3). Three of the five isolates belonged to cgST 8041 from the years 2012 and 2013 (Figure 4). The core AMR genotype of these five isolates comprised resistance genes *bla*_CMY-2_, *aph*(3′′)-Ib, *aph*(6)-Id, *tet*(A), *floR*, and *sul*2. One isolate in this cluster also carried *bla*TEM-1B and *aph*(3′′)-Ia resistance genes (Figure 4A).

The third cluster (branch L) included six isolates, five of which belonged to cgST 200890 from one single dairy herd in 2011, 2014, 2017, and 2021 (Figure 4 and Supplementary Table S2). All isolates in this cluster encoded for *bla*_CMY-2_, *aph*(3′)-Ia, *aph*(3′′)-lb, *aph*(6)-Id, *tet*(A), *floR*, and *sul2* resistance genes. Interestingly, despite the presence of these resistance genes, the isolates in this cluster did not express phenotypic resistance to beta-lactams and aminoglycosides (Figure 3).

The fourth cluster comprised four branches (A, B, E, and F) and exhibited four different phenotypes (P5-P8). This was the most significant cluster, comprising 87 of 109 isolates (80%), with a core antimicrobial resistance profile consisting of resistance to ampicillin, ceftiofur, chlortetracycline, florfenicol, oxytetracycline, and sulfamethazine, with additional resistance to enrofloxacin (branches A and E) and trimethoprim-sulfamethoxazole (branches A and B) (Figure 3A and Figure 4A). The isolates in this cluster belonged to six resistance genotypes, with the core genotype comprising *bla*_CMY-2_, *aph*(3′′)-Ib, *aph*(6)-Id, *tet*(A), *floR*, and *sul2*, also observed in isolates from cluster 2 (Figure 4B).

The fifth cluster included 10 isolates distributed across five branches (D, C, H, J, and G). The underlying antimicrobial resistance profile consisted of resistance to chlortetracycline, florfenicol, oxytetracycline, and sulfadimethoxine, with additional resistance to ampicillin (branches C and G) and trimethoprim-sulfamethoxazole (branches C and D) (Figure 3B). The core resistance genotype comprised *bla*_CMY-2_, *aph* (3′′)-Ib, *aph*(6)-Id, *tet*(A), *floR*, and *sul2* genes (Figure 4A).

### 3.5. Plasmids and Mobile Genetic Elements Associated with AMR Genes

The analysis of plasmid replicons showed that 100 (92%) isolates contained a combination of plasmid replicons of types IncC, IncFII, and IncX1. One isolate had an additional IncX4 replicon, and another had additional IncFIA and IncFIB replicons, while the IncC replicon was missing in six (5.5%) isolates. All 100 isolates that had the IncC plasmid replicon carried the AMR genes *aph*(3′′)-Ib, *aph*(6)-Id, *floR*, *sul*2, *tet*(A). The *bla*_CMY-2_ gene was present in 97 of them, and *aph*(3′)-Ia was present in 16 isolates. Of the six isolates that lacked the IncC replicon, five did not carry any antimicrobial resistance genes, and one lacked *aph*(3′)-Ia and *bla*_CMY-2_.

**Figure 5:**
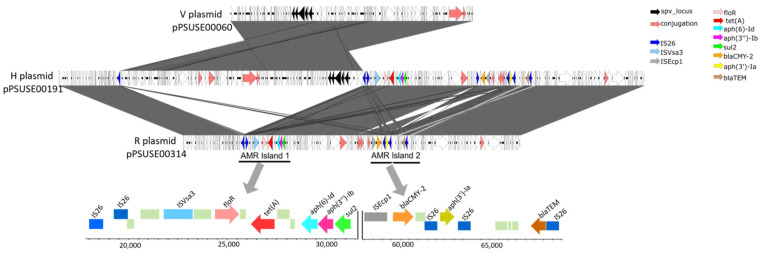
A linear representation of the plasmids observed in this study. The V plasmid pPSUSE00060 is a virulence plasmid that harbors the spv operon. The R plasmid pPSUSE00314 is a resistance plasmid that carries two distinct islands (AMR island 1 and AMR island 2). The two islands are located in a region of the plasmid that is flanked by two IS26 elements. The H plasmid pPSUSE00191 is a hybrid plasmid that includes elements from V and R plasmids.

A linear representation of the plasmids (Figure 5) shows the co-existence of two types of plasmids in the sequenced genomes. The first type is a virulence plasmid with virulence replicons IncX1 and IncFII that harbors the spv operon. The second type is the resistance plasmid with the resistance replicon IncC. In some isolates, a hybrid of both types of plasmids was observed. In these “hybrid” plasmids, the region corresponding to the virulence plasmid is flanked by two IS26 elements. AMR genes are grouped on resistance plasmids in two distinct islands (AMR island 1 and AMR island 2). The two islands are located on a region of the plasmid that is flanked by two IS26 elements. Each of the two islands has an additional specific IS element. The insertion sequence Vsa3 is associated with AMR island 1, which harbors the AMR genes *floR*, *tet*(A), *aph*(6)-Id, *aph*(3′′)-Ib, and *sul2*. ISEcp1 is associated with AMR island 2, which carries the *bla*_CMY-2_ gene. The same island also carries the AMR genes *aph*(3′)-Ia and the truncated version of *bla*TEM-IB, which is missing 37 nucleotides at the 5′ end.

## 4. Discussion

The application of whole genome sequencing, with advanced bioinformatics tools to analyze entire genomes, has led to the development of powerful molecular typing tools. One such tool is cgST, which possesses a high level of discriminatory power. This molecular typing system is widely utilized for prospective and retrospective molecular epidemiological investigations. Many public health laboratories in various countries responsible for monitoring animal, human, and foodborne bacterial diseases have adopted the use of cgSTs to classify and group isolates based on their association with the source (individuals/animals/farms/food), geographical location, and time of occurrence [36].

The spatial distribution of cgSTs from Pennsylvania was analyzed by comparing their placement within both geographical clusters of the farms and five identified phylogenetic clusters. A key finding is that the phylogenetic clusters do not correspond to the geographic clusters, as farms within the same region harbored isolates with different genotypes. Additionally, many cgSTs were found to persist on the same farms over several years, highlighting the local distribution and persistence of *S*. Dublin. This localized pattern is further emphasized by the fact that the isolates from Pennsylvania shared genotypes only with those from North America. These findings corroborate the conclusions of Violante et al. [37], who demonstrated that *S.* Dublin exhibits regional persistence and genetic clustering influenced by geographical factors in cattle and raw milk cheese in France. Additionally, Fenske et al. [38] found that *S.* Dublin populations are shaped by geography, with region-specific clades dominating global distribution. These findings align with our study, which indicated that certain cgSTs of *S*. Dublin are more prevalent locally in Pennsylvania, while others are shared across the U.S. and Canada, suggesting regional adaptation and dissemination through animal movement.

The cgSTs (n = 10) in cluster A were observed in veal calves (total of seventeen) from 2011 to 2020, with only five of them also isolated from dairy cattle over the same period. Conversely, cgSTs in cluster B showed a similar distribution between veal and dairy cattle from 2011 to 2021, which could have been influenced by the most predominant cgST 8041. This cgST was found to persist in veal and dairy cattle on farms in the same geographical areas. The cluster C cgSTs showed a different spatial distribution, with the predominance of cgSTs in 2011, followed by infrequent occurrence after 2012. Contrary to what was observed with cgSTs in clusters A, B, and C, the cgSTs in clusters D and E were predominant after 2017 and 2016, respectively. These observations provide new insights into the distribution of *S.* Dublin over time in Pennsylvania. It can be inferred that *S.* Dublin cgSTs can persist on the same veal or dairy farms over multiple years. When an infection repeatedly occurs on the same farm(s) and neighboring cattle farms or in the same regions over a given period, it supports the notion that *S.* Dublin has a high propensity to become endemic [39,40].

The cgSTs observed in Pennsylvania were also observed in 14 other states in the U.S., all of which have extensive veal and dairy operations. This suggests the diversity of the cgSTs observed in veal calves in Pennsylvania. This observation reveals a critical pathway through which new cgSTs are introduced in Pennsylvania.

*S.* Dublin is associated with a higher incidence of respiratory disease in older dairy calves nearing weaning age (210–240 days) [41,42]. This study examined 39 *S*. Dublin-positive cattle necropsied at the Penn State Animal Diagnostic Laboratory from 2011 to 2022, with an average age of 80 days for males and 116 days for females, comparable to previous reports [41,42,43,44]. Our findings revealed that most animals exhibited lesions consistent with pneumonia, along with varying degrees of gastrointestinal involvement, hepatic and splenic necrosis, lymphadenitis, and renal lesions. The histopathologic findings align with those of Pecoraro et al. [43], who reported similar inflammation and necrosis rates in various organs. A proposed histopathology case definition for *S.* Dublin infection in Holstein cattle less than 180 days old includes specific pulmonary, hepatic, splenic, and lymphatic lesions.

Our study exhibited high levels of antimicrobial resistance, particularly to ampicillin, ceftiofur, chlortetracycline, enrofloxacin, florfenicol, oxytetracycline, sulfadimethoxine, and trimethoprim/sulfamethoxazole. These findings align with similar reports from New York [45], showing comparable resistance profiles across *S.* Dublin isolates around the same period (2007–2021). The resistance profiles in our study are also comparable to those reported by Srednik et al. [46], who examined 140 *S.* Dublin isolates from 21 states submitted to the National Veterinary Services Laboratory for serotype determination between 2014 and 2017. They found resistance to sulfonamides, tetracyclines, aminoglycosides, and beta-lactams in 96%, 97%, 95%, and 85% of the isolates, respectively. These high levels of antimicrobial resistance could be attributed to increased therapeutic antimicrobial use on calf ranches that may create selection pressure for resistant strains of *S*. Dublin. There may be increased disease treatments on calf ranches because of the transport of neonatal calves from the dairies to the calf growing operations, the commingling of calves from several dairies, colostrum-deprived calves, suboptimal feeding, and other stressors [47].

Beta-lactamase genes *bla*TEM and *bla*_CMY_ have been extensively reported in *S*. Dublin isolates of bovine origin. Multiple studies conducted in the USA and Canada [46,48,49,50] reported the presence of *bla*_CMY-2_ with frequencies ranging from 58% to 100%, while frequencies ranging from 5.8 to 80% were reported for variants of *bla*TEM. However, the *bla*TEM-IB gene has a 37-nucleotide truncation at the 5′ end of the gene in all isolates that carried it, which is believed to be caused by the replicative action of an insertion sequence IS26 transposon [13]. Since all these isolates also carried the *bla*_CMY-2_ gene, it can be inferred that the observed resistance to beta-lactam antibiotics is mediated by *bla*_CMY-2_. In summary, based on previous reports and our findings, it can be assumed that *bla*_CMY-2_ is present in a high proportion of *S.* Dublin isolates. This observation poses a significant public health concern, as *bla*_CMY-2_ confers resistance to amoxicillin-clavulanic acid, ampicillin, cefoxitin, ceftiofur, and ceftriaxone.

In our study, resistance to tetracycline was associated with the presence of *tet*(A), the most prevalent of the two *tet* genes (*tet*A and *tet*B). Previous studies have reported it in 95–97% of *S*. Dublin isolates [46,48,49]. Resistance to aminoglycosides is conferred by aminoglycoside O-phosphotransferases (APHs), particularly the subclass *aph*(3′)-I [51]. In our study, *aph*(3′)-Ia and *aph*(3′′)-Ib, which confer resistance to aminoglycosides such as kanamycin, were observed in 15% and 95% of the isolates, respectively. Additionally, the *aph*(6)-Id gene, which is carried on a broad host range of multicopy plasmids that can replicate in most Gram-negative bacteria, was detected in 95% of the isolates. These findings are consistent with other studies that analyzed *S*. Dublin isolates from cattle, humans, and beef [9,48,50,52]. None of the isolates in our study were found to be resistant to gentamicin. Resistance to gentamicin is conferred primarily by aac(3)-1 variants and *aph*(2”)-1 genes [51]. Neither of these genes were found in our isolates, which explains the sensitivity to gentamicin observed.

In the dairy industry, florfenicol, a phenicol group of antimicrobials, is commonly used to treat primarily respiratory infections in calves and non-lactating cattle under 20 months of age. Resistance to florfenicol was first observed in a multi-drug-resistant strain of *S*. Typhimurium DT104. Since then, the resistance to florfenicol among various Salmonella serotypes, including *S*. Dublin, from food animal species, has significantly increased. In our study, 95% of *S*. Dublin isolates carried the *floR* gene, which has also been shown to occur in more than 90% of *S*. Dublin isolates from cattle, humans, and beef in the United States [46,47,48,50].

Resistance to folate pathway inhibitors, such as sulfonamides, is encoded by *sul* genes, which are widely carried by Gram-negative bacteria, including various serotypes of *Salmonella*. In this study, *sul2* was the only *sul* gene detected in 95% of *S*. Dublin isolates. This is consistent with previous reports on *S*. Dublin isolates from humans in Pennsylvania [50] and in cattle and beef samples [13,46,48,49]. *S.* Resistance to quinolones such as enrofloxacin is facilitated by mutated bacterial type II topoisomerases such as DNA gyrases (*gyrA* and *gyrB*) [53]. In our study, we observed *gyrA*_D87Y (2%), *gyrA*_S83F (3%), and *gyrA*_S83Y (5%) mutations in the *gyrA* gene. The study conducted by the National Veterinary Services Laboratory observed five variants of the *gyrA* gene, with *gyrA*_D87Y, *gyrA*_S83F, and *gyrA*_S83Y mutations observed in 36%, 25%, and 7% of *S.* Dublin isolates, respectively [46]. Of the three mutations observed in our study, *gyrA*-S83F was also observed in nine nalidixic-acid-resistant isolates of *S.* Dublin from cattle in Arizona in 2003 [48] and from six quinolone-resistant isolates from a study in Canada [13]. Other mutations, such as *gyrA*-87N and *gyrA*-D87G, have been reported in *S*. Dublin isolates from cattle in the United States and Canada and human isolates from Pennsylvania [50]. However, these two specific mutations were not observed in our cattle isolates from Pennsylvania. The absence of these mutations in *S.* Dublin strains could be due to regional antimicrobial selection pressures and genetic backgrounds. Agricultural practices and antimicrobial usage in Pennsylvania may not have been strong enough to cause these mutations in bovine isolates.

All acquired antimicrobial resistance genes identified in this study were carried on resistance plasmids of the IncC type. These conjugative plasmids are known to mediate the mobilization of antimicrobial resistance determinants, such as the Integrative Antibiotic Resistance Element SGI1, as described by Pons et al. [54]. The plasmids with replicon type IncC, historically labeled IncC/IncA or IncA/C, were observed in multi-drug isolates of several *Salmonella* serotypes [55].

MGEs, such as transposons, play a significant role in transferring and disseminating AMR genes. In this study, multiple insertion sequence (IS) elements were identified. Notably, multiple IS26 elements were associated with both AMR islands on the resistance plasmids, and their position seems to indicate that they are responsible for the transposition of these regions. This is not surprising, as IS26, a member of the IS6 family of insertion sequences, is one of the most widespread transposable elements in multi-drug-resistant bacteria [56]. It is also very likely that IS26 elements contributed to the formation of the virulence-resistance hybrid plasmids observed in some of our isolates. Similar instances of IS26-mediated “fusion” of virulence and resistance plasmids have been reported. Examples include the pN13-01125 plasmid observed in *S.* Dublin by Mangat et al. [57] and plasmid pUO-SeVR1 observed in S. Enteritidis by Garcia et al. [58].

Although those plasmids share many antimicrobial resistance genes, there are variations in the patterns of AMR genes. This variation may be attributed, at least partly, to the differences in the distribution of additional transposable elements that may be associated with specific AMR genes. In this study, the insertion sequence Ecp1 was part of the AMR island 2, directly upstream of the AMR gene *bla*_CMY-2._ The association of ISEcp1 and bacterial resistance with extended-spectrum beta-lactam antibiotics is well documented. It is believed that the ISEcp1 is responsible for capturing and mobilizing various types of extended-spectrum beta-lactamase genes, such as *bla*_CTX-M_ [59,60] and *bla*_CMY-2_ [61]. ISVsa3 was found in AMR island 1 in association with AMR genes *floR*, *tet*(A), *aph*(6)-Id, *aph*(3′′)-Ib, *aph*(3′)-Ia, and *sul2*. A similar configuration was observed on *S.* Dublin plasmid pRM104_1 (GenBank accession CP117347), as reported by Lewis et al. [62]. The combination of the conjugative resistance IncC plasmids and various transposable elements observed in this study is yet another demonstration of bacterial molecular tools for the horizontal transfer of antimicrobial resistance.

Our findings align with previous studies demonstrating the regional persistence and antimicrobial resistance of *S.* Dublin in dairy cattle [37,38]. The high prevalence of MDR strains in Pennsylvania, particularly resistance to beta-lactams and enrofloxacin, emphasized the phenotypic role in *S*. Dublin. To mitigate these risks, policy interventions should focus on enhancing biosecurity protocols, regulating calf movement, and implementing antimicrobial stewardship programs to limit resistance development. Additionally, integrating WGS can provide early outbreak detection and improve tracking of resistant strains, aiding in targeted interventions to control *S.* Dublin in dairy and veal operations.

## 5. Conclusions

Our study highlights the geospatial distribution and antimicrobial resistance of *S.* Dublin in Pennsylvania, emphasizing its public health and agricultural implications. The high levels of multidrug resistance (MDR), particularly to beta-lactams, tetracyclines, and enrofloxacin, complicate treatment and raise concerns about zoonotic transmission through direct contact, contaminated meat, raw milk, and environmental pathways. The regional prevalence of distinct cgSTs suggests that calf movement plays a key role in dissemination, reinforcing the need for enhanced farm biosecurity measures, stricter antimicrobial stewardship, and targeted surveillance programs to mitigate the spread and impact of *S.* Dublin in dairy and veal operations.

## Figures and Tables

**Table 1:** Pathological findings on male and female calves submitted for necropsy at the Penn State Animal Diagnostic Laboratory with confirmed *Salmonella* Dublin Infection (2011–2021).

System	Lesions	Male (n = 18; 14 Farms/11 Years)		Female (n = 21, 13 Farms/9 Years)	
		Lesions (%)	Total by System (%)	Lesions (%)	Total by System (%)
Respiratory	Bronchopneumonia	34	89	24	95
	Interstitial pneumonia	28		29	
	Mixed pneumonia	28		43	
Gastro-intestinal	Abomasitis	5.5	61	14	80
	Abomasal ulcer	5.5		5	
	Enteritis	50		52	
	Peritonitis	11		5	
	Rumenitis	-		5	
Hepatic	Hepatic necrosis	50	55.5	61	61
	Hepatomegaly	5.5		-	
Lymphatic	Splenic necrosis	22	50	24	43
	Lymphadenitis	39		24	
Urinary	Tubulointerstitial nephritis	17	28	19	28
	Interstitial nephritis	11		10	
Cardiovascular	Pericardial effusion	5.5	5.5	-	-
	Cardiomyocyte degeneration	-	-	5	5
Reproductive	Placental necrosis	-	-	5	5
Age: Mean, median, (range)		80, 59, (14–252)		116, 84, (21–630)	

**Table 2:** Antimicrobial resistance of *Salmonella* Dublin isolates from veal and dairy cattle in Pennsylvania from 2011 to 2022.

Antimicrobial	Resistance Breakpoints ^a,b^	Antimicrobial Concentrations (µg/mL) ^c^															Antimicrobial Resistance Genes
		<0.12	<0.25	0.5	<1	1	<2	2	>2	4	8	>8	16	>16	<256	>256	
		% Isolates (n = 109)															
Ampicillin	≥32 ^a,b^			2		6		5						87 ^c^			*bla*_CMY-2_ (92%) *bla*_TEM-1B_ (25%)
Ceftiofur	≥4 ^b^			6		5					8 ^c^	81 ^c^					
Chlortetracycline	≥16 ^a,b^					5		1				94 ^c^					*tet*(A) (96%)
Oxytetracycline	≥16 ^a^					4		2				94 ^c^					
Enrofloxacin	≥0.12 ^a^	83	2 ^c^	5 ^c^		7 ^c^		3 ^c^									*gyrA*_D87Y (2%) *gyrA*_S83F (3%) *gyrA*_S83Y (5%)
Florfenicol	≥16 ^a,b^					1		3		2		94 ^c^					*floR* (96%)
Gentamicin	≥16 ^b^				96			2		2							*aph*(3′)-Ia (15%) *aph*(3′′)-Ib (96%) *aph*(6)-Id 96%)
Sulfadimethoxine	≥512 ^a^														3	97 ^c^	*sul*2 (96%)
Trimethoprim/sulfamethoxazole	≥4 ^a^						80		20 ^c^								

^a^ CLSI M100-31st ed. (2021) [18] and ^b^ CLSI Supplement VET08 (2021) [35]. ^c^ Percentages of resistant isolates (with MIC values above resistance breakpoints).

## Data Availability

The WGS data were submitted to the Sequence Read Archive of the National Center for Biotechnology Information under the BioProject number PRJNA1109834.

## Supplementary Materials

The following supporting information can be downloaded at https://www.mdpi.com/article/10.3390/microorganisms13020400/s1. Supplementary Table S1: Core-genome sequence types (cgSTs) of *Salmonella* Dublin; metadata associated with corresponding genomics data retrieved from the NCBI SRA database. Supplementary Table S2: *S.* Dublin cgSTs identified at the Penn State Animal Diagnostic Laboratory (2011–2022) from veal and dairy calves in Pennsylvania.

## Abbreviations

The following abbreviations are used in this manuscript:

cgST	Core-genome sequence types
MDR	Multidrug-resistant
WGS	Whole genome sequencing
MIC	Minimum inhibitory concentrations
SISTR	*Salmonella* in silico typing resource
MGEs	Mobile genetic elements
