# Supplementary material for: Molecular Epidemiology of Salmonella enterica Serotype Dublin Isolated from 2011 to 2022 from Veal and Dairy Cattle in Pennsylvania
Source: Microorganisms. 2025 Feb 12;13(2):400. doi: 10.3390/microorganisms13020400 (PMC11858433; doi:10.3390/microorganisms13020400)
Supplement: Supplementary file 1 [file microorganisms-13-00400-s001.zip › Supplementary Table S1.pdf]

**Supplementary Table S1.** Core-genome sequence types (cgSTs) of *Salmonella* Dublin in the USA and Canada, metadata associated with corresponding genomics data retrieved from the NCBI SRA database.

| cgST* | Phylo_Cluster | USA and Canada | Year | SRA Accession Number | Sources |
|---|---|---|---|---|---|
| 70980 | A | NY | 2016 (1) | SRR4292612 | intestine (Bos taurus) |
| 70980 | A | NY | 2017 (1) | SRR5576108 | product-raw-intact-beef |
| 81805 | A | IA | 2015 (2) | SRR5905275, SRR5905281 | lung (Bos taurus) |
| 81805 | A | WI | 2022 (1) | SRR27714938 | lung (Bos taurus) |
| 98761 | A | - | - | - | - |
| 98901 | A | - | - | - | - |
| 111266 | A | WI | 2023 (1) | SRR24263593 | product-raw-intact-beef |
| 116668 | A | OH | 2012 (1) | SRR7011732 | feces (Bos taurus) |
| 116668 | A | OH | 2015 (2) | SRR7041722, SRR7041896 | lung (Bos taurus) |
| 116668 | A | WI | 2023 (1) | SRR27187897 | comminuted beef |
| 116820 | A | - | - | - | - |
| 117842 | A | USA† | 2018 (1) | SRR7049822 | blood |
| 117842 | A | WI | 2022 (1) | SRR22485980 | comminuted beef |
| 117842 | A | NY | 2023 (1) | SRR24980046 | farm environment |
| 117842 | A | Canada | 2023 (1) | SRR26904505 | liver (Bos taurus) |
| 123218 | A | OH | 2014 (1) | SRR7221730 | lung (Bos taurus) |
| 125181 | A | CO | 2015 (1) | SRR1770503 | dog treat |
| 125181 | A | OH | 2017 (1) | SRR6278772 | pooled tissue (Bos taurus) |
| 125181 | A | WV | 2023 (1) | SRR23878024 | ground beef |
| 8041 | B | NY | 2010 (1) | SRR3097505 | feces (Bos taurus) |
| 8041 | B | NY | 2011 (3) | SRR2994390, SRR2994390, SRR3000884 | feces (Bos taurus) |
| 8041 | B | NY | 2011 (5) | SRR3037403, SRR3000888, SRR2994392, SRR3037520, SRR3000891 | lung (Bos taurus) |
| 8041 | B | NY | 2011 (1) | SRR3000887 | lymph node (Bos taurus) |
| 8041 | B | NY | 2011 (1) | SRR2994395 | kidney (Bos taurus) |
| 8041 | B | NY | 2011 (1) | SRR3037518 | intestine (Bos taurus) |
| 8041 | B | NY | 2011 (1) | SRR3000886 | caecum (Bos taurus) |
| 8041 | B | NY | 2012 (2) | SRR3037400, SRR3037467 | feces (Bos taurus) |
| 8041 | B | IA | 2015 (1) | SRR5928634 | lung (Bos taurus) |
| 8041 | B | IA | 2016 (1) | SRR5928135 | lung (Bos taurus) |
| 8041 | B | PA | 2024 (1) | SRR29789981 | comminuted beef |

| | | | | | |
|---|---|---|---|---|---|
| 8041 | B | OH | 2023 (1) | SRR23677017 | product-raw-intact-beef |
| 8041 | B | Canada | 2022 (1) | SRR24907574 | liver (Bos taurus) |
| 8041 | B | Canada | 2022 (1) | SRR24146401 | lung (Bos taurus) |
| 8169 | B | NY | 2012 (1) | SRR3037491 | lung (Bos taurus) |
| 8169 | B | NY | 2012 (1) | SRR3037517 | lymph node (Bos taurus) |
| 8311 | B | - | - | - | - |
| 15790 | B | ID | 2019 (1) | SRR10493644 | comminuted beef |
| 15790 | B | ID | 2022 (1) | SRR26124582 | gall bladder (Bos taurus) |
| 15790 | B | ID | 2022 (2) | SRR22381737, SRR22927603 | comminuted beef |
| 15790 | B | ID | 2022 (1) | SRR18297514 | product-raw-intact-beef |
| 15790 | B | WI | 2003 (1) | SRR1593389 | lung (Bos taurus) |
| 15790 | B | WI | 2022 (1) | SRR27714934 | lung (Bos taurus) |
| 15790 | B | WI | 2023 (1) | SRR1284696 | lung (Bos taurus) |
| 15790 | B | MN | 2015 (1) | SRR5928608 | lung (Bos taurus) |
| 15790 | B | MN | 2018 (1) | SRR8270022, SRR8270029, SRR8270017 | comminuted beef |
| 15790 | B | TX | 2021 (1) | SRR16499293 | comminuted beef |
| 15790 | B | TX | 2022 (1) | SRR18319677, SRR22927701 | comminuted beef |
| 15790 | B | TX | 2023 (16) | SRR25475439, SRR25475447, SRR25475444, SRR25727917, SRR25727909, SRR25727916, SRR25727940, SRR25763873, SRR25763872, SRR25763874, SRR26125206, SRR26628117, SRR26628115, SRR26628123, SRR26628124, SRR26628125 | comminuted beef |
| 15790 | B | CA | 2018 (2) | SRR6818876, SRR6818840 | comminuted beef |
| 15790 | B | CA | 2021 (3) | SRR16966794, SRR16966793, SRR16966795 | comminuted beef |
| 15790 | B | CA | 2023 (2) | SRR23686737, SRR16969596 | comminuted beef |
| 15790 | B | CA | 2024 (1) | SRR28871849 | comminuted beef |
| 15790 | B | WA | 2023 (1) | SRR23375234 | product-raw-intact-beef |
| 15790 | B | NE | 2023 (1) | SRR24939835 | product-raw-intact-beef |
| 36016 | B | SD | 2014 (1) | SRR2102453 | colon (Bos taurus) |
| 36016 | B | TN | 2021 (1) | SRR16553250 | comminuted beef |
| 36016 | B | Canada | 2017 (1) | SRR17263709 | Joint fluid (Homo sapiens) |
| 41931 | B | - | - | - | - |
| 49265 | B | - | - | - | - |
| 57144 | B | USA† | 2016 (2) | SRR5341712, SRR5125979 | blood |
| 57144 | B | USA† | 2017 (1) | SRR6283536 | urine |

| | | | | | |
|---|---|---|---|---|---|
| 57144 | B | Canada | 2022 (1) | SRR24146397 | liver (Bos taurus) |
| 57144 | B | Canada | 2023 (1) | SRR26904506 | liver (Bos taurus) |
| 146370 | C | PA | 2022 (1) | SRR18018981 | cecal (veal, bovine) |
| 146370 | C | NE | 2022 (6) | SRR18021225, SRR18128643, SRR18128662, SRR18128661, SRR18128669, SRR18208211 | comminuted beef |
| 146370 | C | TX | 2023 (1) | SRR27563175 | comminuted beef |
| 146370 | C | Canada | 2023 (2) | SRR26904504, SRR28098925 | liver (Bos taurus) |
| 146372 | C | - | - | - | - |
| 146393 | C | - | - | - | - |
| 146413 | C | - | - | - | - |
| 146414 | C | - | - | - | - |
| 147119 | C | - | - | - | - |
| 150213 | C | PA | 2018 (1) | SRR8137073 | feces (Bos taurus) |
| 172182 | D | PA | 2023 (1) | SRR25175891 | farm environment |
| 172182 | D | PA | 2023 (2) | SRR24980048, SRR24980049 | feces (Bos taurus) |
| 172182 | D | PA | 2023 (1) | SRR24980047 | nasal swab (Bos taurus) |
| 180064 | D | MN | 2018 (1) | SRR9016861 | lung (Bos taurus) |
| 180064 | D | OH | 2024 (1) | SRR29455974 | liver (Bos taurus) |
| 180064 | D | PA | 2023 (1) | SRR27891835 | liver (Canis lupus familiaris) |
| 182106 | D | WI | 2019 (1) | SRR9113166 | product-raw-intact-pork |
| 188848 | D | PA | 2023 (1) | SRR29844198 | lung (Bos taurus) |
| 200890 | D | OH | 2019 (1) | SRR10084901 | environmental |
| 204551 | D | NY | 2019 (1) | SRR10240819 | lung (Bos taurus) |
| 204551 | D | NY | 2023 (1) | SRR28830458 | lung (Bos taurus) |
| 229398 | E | NY | 2019 (1) | SRR11440464 | Large intestine (Mus musculus) |
| 229398 | E | WI | 2021 (1) | SRR16541706 | comminuted beef |
| 242215 | E | MN | 2020 (1) | SRR12626352 | comminuted beef |
| 245930 | E | Canada | 2015 (1) | SRR12799638 | stool (Homo sapiens) |
| 259249 | E | PA | 2020 (1) | SRR28995527 | feces (Bos taurus) |

*These cgST were identified in this study of Pennsylvania. Only records with cgSTs identical to those in this study were retained in SRA data.

Rows with missing data indicate cgSTs that were not found in US states and Canada but found in our study in Pennsylvania. †States not found.
