# Supplementary material for: Molecular Epidemiology of Salmonella enterica Serotype Dublin Isolated from 2011 to 2022 from Veal and Dairy Cattle in Pennsylvania
Source: Microorganisms. 2025 Feb 12;13(2):400. doi: 10.3390/microorganisms13020400 (PMC11858433; doi:10.3390/microorganisms13020400)
Supplement: Supplementary file 1 [file microorganisms-13-00400-s001.zip › Supplementary Table S2.pdf]

Supplementary Table S2. *Salmonella* Dublin cgSTs identified at the Penn State Animal Diagnostic Laboratory on veal and dairy calves in Pennsylvania between 2011 to 2022.

cgST (n=35)	No. of Isolates	No. of Farms (Cluster)*	No. of Veal calves	No. of Dairy cattle	No. of Counties	Year(s)
70980	1	1 (A)	1	-	1	16
81805	1	1 (A)	1	-	1	18
98761	2	2 (A)	1	1	2	18, 19
98901	1	1 (A)	1	-	1	20
111266	5	5 (A)	3	2	4	16,17,18,19,20
116668	1	1 (A)	1	-	1	16
116820	6	3 (A)	2	1	3	11,13,14,16
117842	6	5 (A)	4	1	4	19, 20
123218	1	1 (A)	1	-	1	14
125181	2	2 (A)	2	-	2	12, 14
8041	23	18 (B)	13	5	12	11, 12, 13, 14, 15,16,18,19, 20,21
8169	1	1 (B)	1	-	1	16
8311	1	1 (B)	1	-	1	12
15790	5	4 (B)	3	1	2	11,12,14,16
36016	1	1(B)	1	-	1	14
41931	1	1 (B)	1	-	1	11
49265	2	1 (B)	1	-	1	15
57144	8	6 (B)	5	1	4	14,16,17,19,21
146370	4	4 (C)	3	1	4	11
146372	1	1 (C)	1	-	1	11
146393	2	2 (C)	1	1	2	12, 13
146413	1	1 (C)	1	-	1	11
146414	2	2 (C)	2	-	2	11, 12
147119	1	1 (C)	1	-	1	16
150213	1	1 (C)	-	1	1	20
172182	5	2 (D)	1	1	2	18,20,22
180064	2	2 (D)	2	-	2	21
182106	1	1 (D)	1	-	1	19
188848	7	4 (D)	2	2	3	18, 19, 20, 21
200890	5	1(D)	-	1	1	11,14,17,21
204551	1	1(D)	-	1	1	21
229398	1	1(E)	1	-	1	17
242215	2	2 (E)	2	-	2	20
245930	1	1 (E)	1	-	1	16
259249	4	3 (E)	2	2	2	19,20,21

* Phylo-clusters A-E (Figure 1)
** The cgSTs observed in other states were identified through the NCBI sequence read accession (SRA) database (https://www.ncbi.nlm.nih.gov/sra, accessed on August 19, 2024). The metadata associated with the SRA can be accessed through the BioSample links associated with each SRA record.
